# Benzophenone-3 promotion of mammary tumorigenesis is diet-dependent

**DOI:** 10.18632/oncotarget.27831

**Published:** 2020-12-01

**Authors:** Anastasia Kariagina, Elena Morozova, Reyhane Hoshyar, Mark D. Aupperlee, Mitchell A. Borin, Sandra Z. Haslam, Richard C. Schwartz

**Affiliations:** ^1^Breast Cancer and the Environment Research Program, Department of Microbiology and Molecular Genetics, Michigan State University, East Lansing, MI, USA; ^2^Breast Cancer and the Environment Research Program, Department of Physiology, Michigan State University, East Lansing, MI, USA

**Keywords:** oxybenzone, benzophenone-3, mammary tumorigenesis, dietary animal fat, breast cancer

## Abstract

Benzophenone-3 is a putative endocrine disrupting chemical and common ingredient in sunscreens. The potential of endocrine disrupting chemicals to act as agonists or antagonists in critical hormonally regulated processes, such as mammary gland development and mammary tumorigenesis, demands evaluation of its potential in promoting breast cancer. This study identifies the effects of BP-3 on mammary tumorigenesis with high-fat diet during puberty versus adulthood in *Trp53*-*null* transplant BALB/c mice. Benzophenone-3 exposure yielded levels in urine similar to humans subjected to heavy topical sunscreen exposure. Benzophenone-3 was protective for epithelial tumorigenesis in mice fed lifelong low-fat diet, while promotional for epithelial tumorigenesis in mice fed adult high-fat diet. Benzophenone-3 increased tumor cell proliferation, decreased tumor cell apoptosis, and increased tumor vascularity dependent on specific dietary regimen and tumor histopathology. Even in instances of an ostensibly protective effect, other parameters suggest greater risk. Although benzophenone-3 seemed protective on low-fat diet, spindle cell tumors arising in these mice showed increased proliferation and decreased apoptosis. This points to a need for further studies of benzophenone-3 in both animal models and humans as a potential breast cancer risk factor, as well as a more general need to evaluate endocrine disrupting chemicals in varying dietary contexts.

## INTRODUCTION

Ovarian hormones are strongly implicated in the etiology of breast cancer [1, 2], and are particularly important for the development of the breast during puberty and young adulthood [3]. Putative endocrine disrupting chemicals (EDCs), particularly estrogenic chemicals, have emerged as suspects in environmental promotion of breast cancer [4]. Environmental EDCs have the potential to act as agonists or antagonists in critical hormonally regulated processes, such as mammary gland development and mammary tumorigenesis. This warrants evaluation of their potential in promoting breast cancer. We demonstrated enhancement of mammary tumorigenesis by a diet high in saturated animal fat (HFD) [5–8]. Thus, examination of the activity of EDCs in a dietary context may provide additional insight into the potential role of EDCs in promoting breast cancer.

Benzophenone-3 (BP-3; oxybenzone) is a putative EDC and a common active ingredient in sunscreens and other personal care products [9]. Beyond direct exposure by these products, BP-3 is present in household dust [10], in fish lipids [11], and, notably, in the aqueous environment [9]. More recently, BP-3 was demonstrated to have pathological effects on coral [12]. Although BP-3 has a very short half-life, its presence is widespread in human urine [9], in as much as 98% of the general U.S. population [13]. A recent preliminary study found plasma concentrations greater than 0.5 ng/mL among a small human cohort using heavy topical applications of commercial sunscreens. This level, achieved after only one d exposure, exceeds Food and Drug Administration guidance for chemicals of “threshold of toxicological concern” [14].

BP-3 has known estrogenic and anti-estrogenic properties [9]. We have shown that both estrogen [15, 16] and HFD [17, 5] can modulate proliferative, inflammatory and angiogenic activity in the mammary gland. Thus, it is logical to test their individual and combined effects on mammary tumorigenesis. The present study examined the interaction of BP-3 with HFD on mammary tumorigenesis in BALB/c mice, using the *Trp53*-*null* transplantation model [18]. A level of BP-3 exposure was used that yielded levels in murine urine similar to that observed in humans subjected to heavy topical exposure of BP-3-containing commercial sunscreen [19]. We found that BP-3 had complex effects that were dependent upon dietary regimen and tumor histopathology. BP-3 was protective in regard to epithelial tumor promotion in mice fed a low fat diet (LFD) and was promotional for epithelial tumorigenesis in mice fed HFD restricted to adulthood. At the same time, BP-3 increased tumor cell proliferation, decreased tumor cell apoptosis, and increased tumor vascularity in a manner dependent on specific dietary regimen and tumor histopathology. Increased mammary tumor-free survival was not always concordant with a decrease in properties associated with tumor progression. Notably, although BP-3 seemed protective for tumorigenesis in mice fed LFD, the spindle cell tumors that arose in these mice showed increased proliferation and decreased apoptosis. Collectively, these findings suggest that BP-3 exposure may have adverse consequences in mammary tumorigenesis.

## RESULTS

### BP-3 enhances estrogen-stimulated mammary gland proliferation in pubertal mice fed HFD

**Figure 1:**
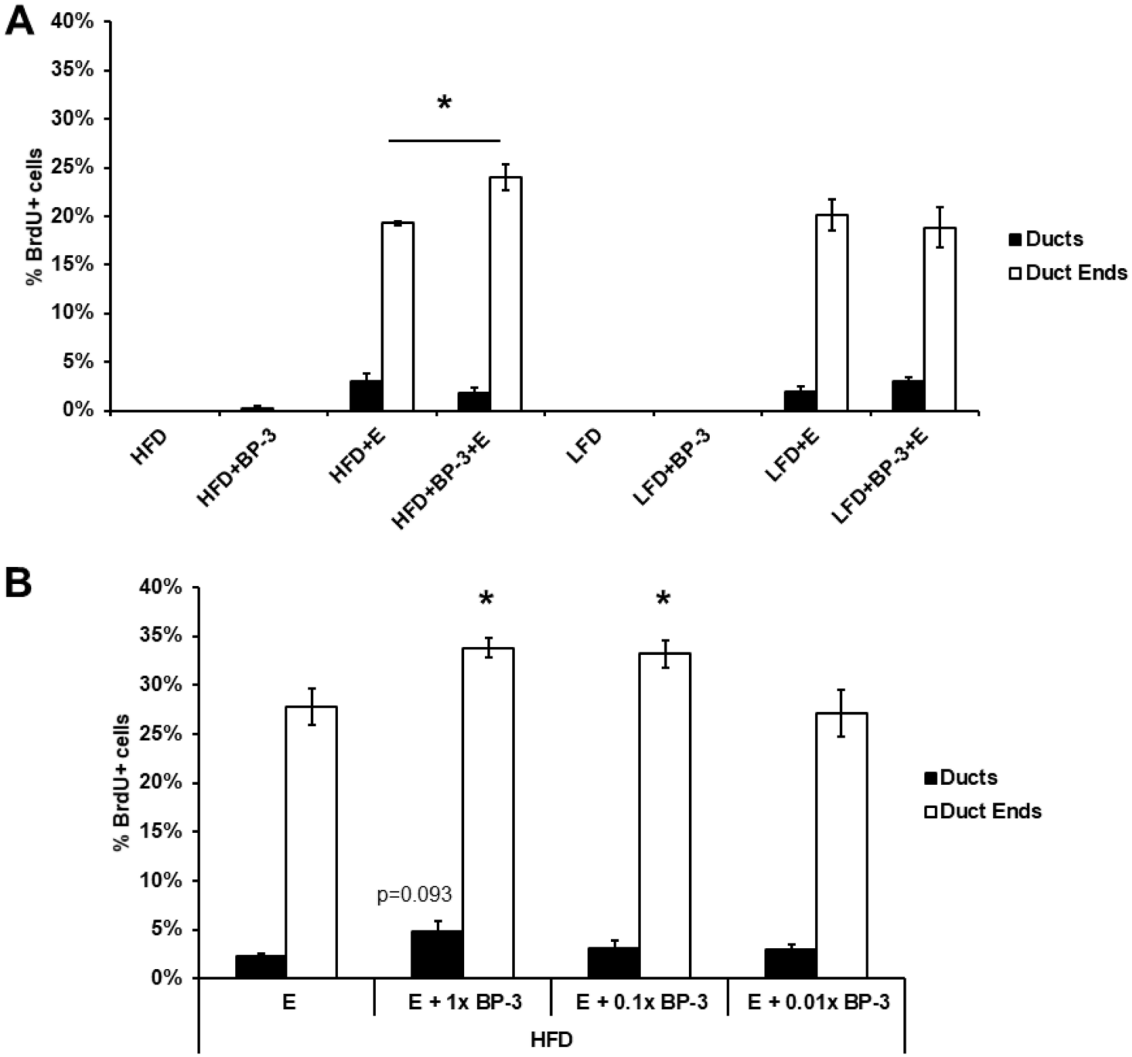
BP-3 enhances estrogen-stimulated mammary gland proliferation in pubertal mice fed HFD. (**A**) Pubertal OVX BALB/c mice were placed on LFD or HFD with and without BP-3, and then treated with E2 or control for 5 d. The pubertal mice fed HFD plus BP-3 showed higher proliferation in response to E2 than did mice fed HFD alone. (**B**) Pubertal OVX BALB/c mice BP-3 were placed on HFD and treated for 5 d with E2 (E) or E2 + BP-3 (1.0×, 0.1×, 0.01× 70 mg/kg BW). BP-3 augmented the proliferative response to E2 in both ducts and duct ends at the standard dose and in duct ends at the 0.1 dose. The values presented are means +/– SEM. Significance of differences between samples was assessed using an unpaired two-tailed Student’s *t*-test. ^*^ *p* < 0.05.

Since BP-3 showed estrogenic activity as a proliferative stimulus to MCF-7 breast cancer cells [20, 21], we sought to see if BP-3 could stimulate *in vivo* mammary epithelial proliferation. To that end, both pubertal and adult BALB/c mice were placed on LFD or HFD with and without BP-3 (see Materials and Methods), ovariectomized (OVX), allowed time for recovery and clearance of endogenous hormones, and then treated with E2 or control for 5 d. While no BP-3 effects were seen in the adult mice (data not shown), the pubertal mice fed HFD plus BP-3 showed higher proliferation in response to E2 than did mice fed HFD alone (Figure 1A). No BP-3 effects were observed in the absence of E2, and no BP-3 effects were observed in mice fed LFD.

As the initial experiment involved relatively long-term treatment with BP-3 and acute exposure to E2, we examined the effects of BP-3 in an acute exposure regimen at our standard dose, as well as 0.1 and 0.01 doses, with and without co-treatment with E2 in OVX pubertal BALB/c mice fed HFD. While BP-3 by itself showed no effects at any dose (data not shown), BP-3 augmented the proliferative response to E2 in both ducts and duct ends at the standard dose and in duct ends at the 0.1 dose (Figure 1B).

### BP-3 reduced tumorigenesis in mice fed LFD and promoted tumorigenesis in mice fed LFD-HFD

**Figure 2:**
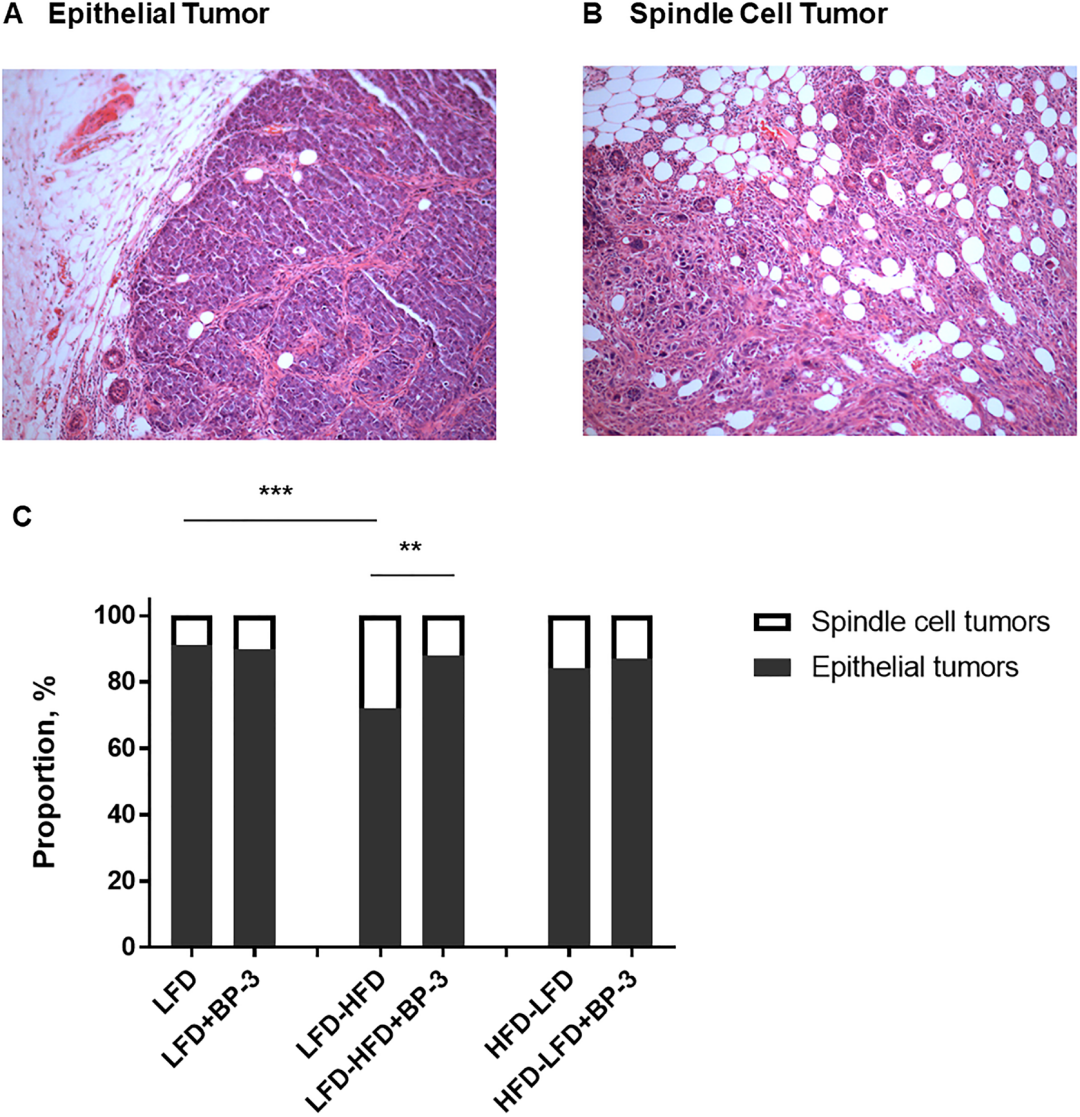
BP-3 increased the proportion of epithelial tumors in mice fed an adult-restricted HFD. (**A**) Representative H&E stained tissue section of an epithelial tumor. Magnification is 10×. (**B**) Representative H&E stained tissue section of a spindle cell tumor. Magnification is 10×. (**C**) Proportion of epithelial and spindle cell tumors across dietary and BP-3 treatments. Significance of differences between tumor groups was assessed using Fisher’s Exact Test. ^**^ *p* < 0.01; ^***^ *p* < 0.001.

Having observed growth promoting effects, we examined whether BP-3 treatment would promote tumorigenesis in the *Trp53-null* mammary transplant model in BALB/c mice. We previously reported that HFD exposure at either puberty or adulthood promoted mammary tumorigenesis in this model [7]. Since our examination of BP-3 stimulated growth in the mammary gland only found effects at puberty with mice fed HFD, we examined the effects of BP-3 on tumorigenesis with mice fed LFD, pubertally-restricted HFD (HFD-LFD), and adulthood-restricted HFD (LFD-HFD). As in our earlier studies, most tumors were epithelial in composition (Figure 2A), while some were poorly differentiated spindle cell carcinomas (Figure 2B). While an adult-restricted HFD (LFD-HFD) increased the proportion of spindle cell tumors compared to LFD, the proportion of epithelial versus spindle cell tumors was increased by BP-3 treatment in mice fed LFD-HFD (Figure 2C).

**Figure 3:**
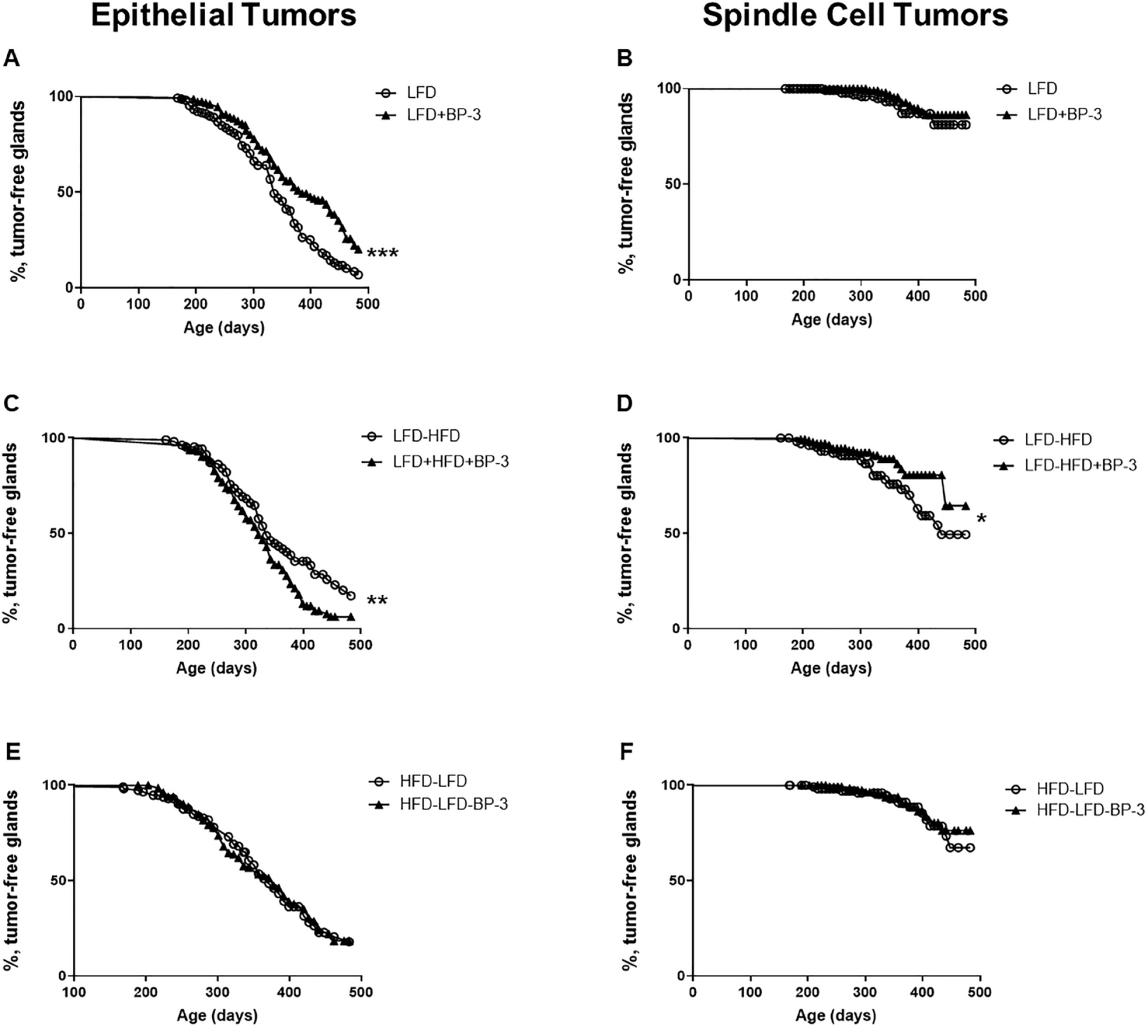
BP-3 reduced epithelial tumorigenesis on LFD, but promoted epithelial tumorigenesis on an adult-restricted HFD. Kaplan-Meier plots were determined for BALB/c mice receiving *Trp53-null* mammary transplants and fed a lifelong LFD (LFD; *n* = 170 glands; 99 mice), a lifelong LFD plus lifelong BP-3 (LFD+BP-3; *n* = 152 glands; 89 mice), an adult-restricted HFD (LFD-HFD, *n* = 108 gland; 65 mice), an adult-restricted HFD plus lifelong BP-3 (LFD-HFD+BP-3, *n* = 147 glands; 82 mice), a puberty-restricted HFD (HFD-LFD, *n* = 115 glands; 66 mice), and a puberty-restricted HFD plus lifelong BP-3 (HFD-LFD, *n* = 136 glands; 78 mice). The number of analyzed glands is less than half the number of mice because of failed transplantation in one gland or the death of some mice after the first tumor had been removed. (**A**) LFD versus LFD+BP-3; epithelial tumors. (**B**) LFD versus LFD+BP-3; spindle cell tumors. (**C**) LFD-HFD versus LFD-HFD+BP-3; epithelial tumors. (**D**) LFD-HFD versus LFD-HFD+BP-3; spindle cell tumors. (**E**) HFD-LFD versus HFD-LFD+BP-3; epithelial tumors. (**F**) HFD-LFD versus HFD-LFD+BP-3; spindle cell tumors. Significance of differences between plots was assessed by the log-rank Mantel–Cox test. ^*^ *p* < 0.05; ^**^ *p* < 0.01; ^***^ *p* < 0.001.

Kaplan-Meier analysis revealed that BP-3 reduced tumorigenesis of epithelial tumors in mice fed LFD (Figure 3A). On the other hand, consistent with the increased proportion of epithelial tumors, BP-3 was promotional for epithelial tumorigenesis in mice fed LFD-HFD (Figure 3C), while reducing spindle cell tumorigenesis (Figure 3D). No significant effects were observed with Kaplan-Meier analysis for either spindle cell tumors in mice fed LFD (Figure 3B), or for both epithelial and spindle cell tumors from mice fed HFD-LFD (Figure 3E and 3F). Total tumor incidence was not significantly altered by BP-3 in any of the dietary regimens on analysis by Fisher’s exact test (data not shown).

**Figure 4:**
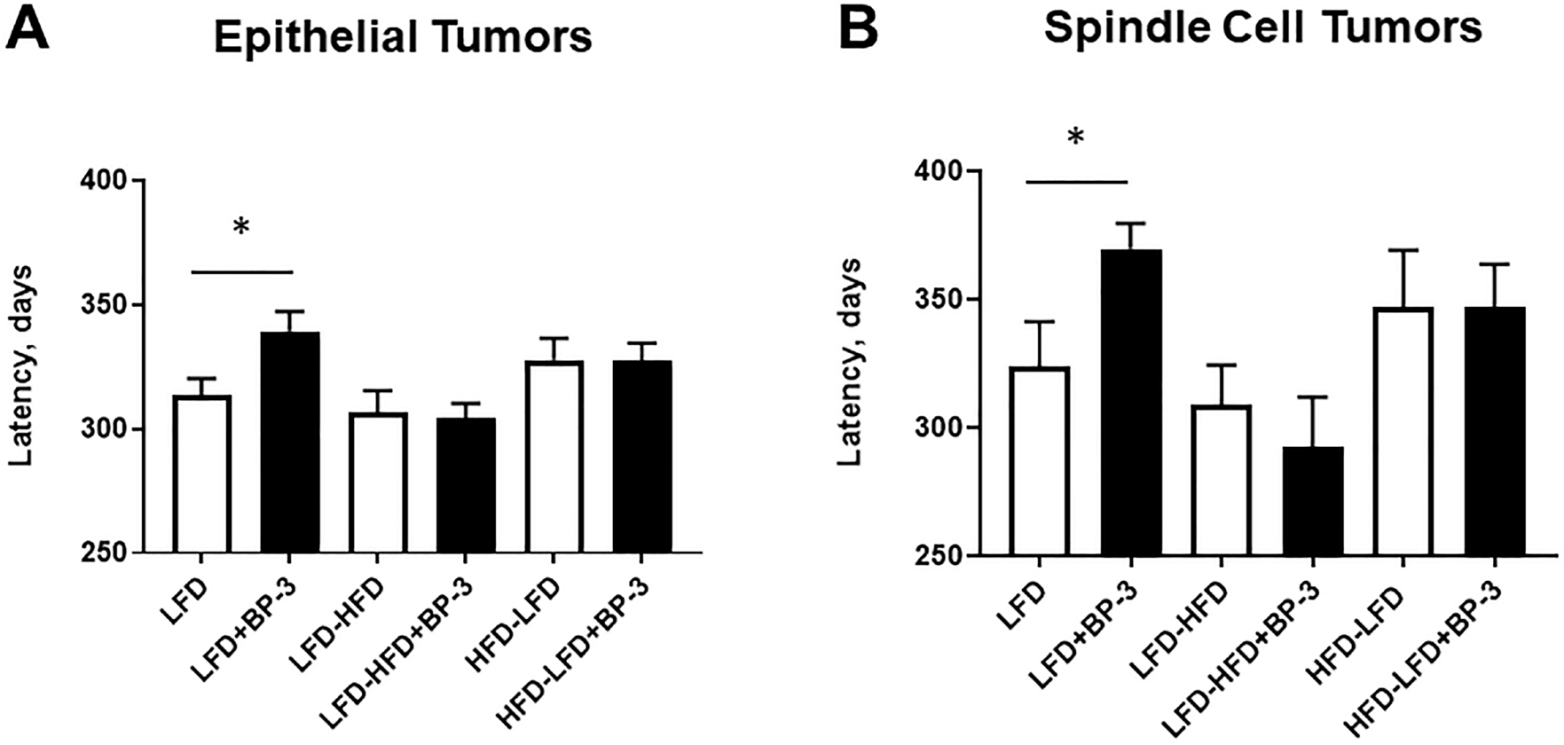
BP-3 treatment increased latency of both epithelial and spindle cell tumors in mice fed LFD. Mean latency of epithelial tumors (**A**) and spindle cell tumors (**B**) are compared by dietary and BP-3 treatment. Numbers of tumors per treatment group: LFD: 116 epithelial, 11 spindle cell; LFD+BP-3: 94 epithelial, 10 spindle; LFD-HFD: 63 epithelial, 25 spindle cell; LFD-HFD+BP-3: 107 epithelial, 15 spindle cell; HFD-LFD: 70 epithelial; 13 spindle cell; HFD-LFD+BP-3: 84 epithelial, 13 spindle cell. The values presented are means +/– SEM. Significance of differences between treatment groups was assessed using the Mann-Whitney *U* test. ^*^ *p* < 0.05.

BP-3 treatment increased latency of both epithelial and spindle cell tumors in mice fed LFD (Figure 4A and 4B). No significant effects on latency were found for BP-3 treatment on other diets. ANOVA found significance for dietary effects in both epithelial and spindle cell tumors (Supplementary Table 1). This is consistent with mice fed LFD-HFD tending to have shorter tumor latencies than mice fed either LFD or HFD-LFD, although this trend is not significant by Mann-Whitney *U* test.

### Tumor characteristics

Most of the epithelial tumors were ER- PR-, ranging from 89 to 100% among the dietary regimens and BP-3 treatments, and did not vary significantly by histological type, diet, or BP-3 treatment. Similarly, most spindle cell tumors were ER- PR-, ranging from 88 to 100% among the dietary regimens and BP-3 treatment (Supplementary Table 2).

**Figure 5:**
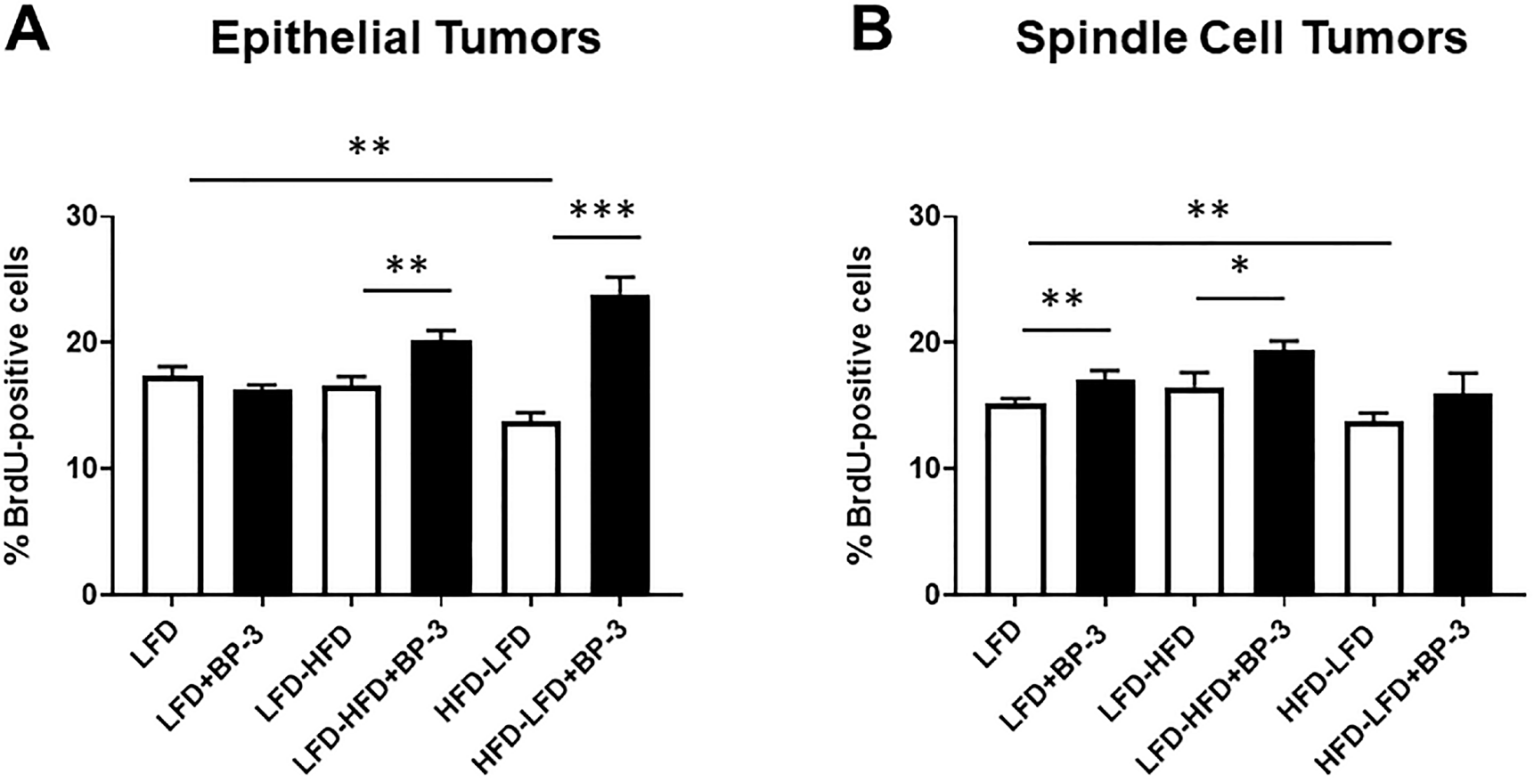
BP-3 treatment increased tumor proliferation in a manner dependent on both diet and histological type. Tumors are grouped by epithelial (**A**) and spindle cell (**B**) histology. Epithelial tumors: LFD (*n* = 8); LFD+BP-3 (*n* = 10); LFD-HFD (*n* = 7); LHF-HFD+BP-3 (*n* = 7); HFD-LFD (*n* = 10); HFD-LFD+BP-3 (*n* = 13). Spindle cell tumors: LFD (*n* = 8); LFD+BP-3 (*n* = 6); LFD-HFD (*n* = 8); LFD-HFD+BP-3 (*n* = 8); HFD-LFD (*n* = 9); HFD-LFD+BP-3 (*n* = 10). The values presented are means +/– SEM. Significance of differences between samples was assessed using an unpaired two-tailed Student’s *t*-test. ^*^ *p* < 0.05; ^**^ *p* < 0.01; ^***^ *p* < 0.001.

Since unregulated proliferation and resistance to apoptosis are hallmarks of cancer, we measured tumor cell proliferation and apoptosis by quantifying BrdU incorporation and TUNEL, respectively. Epithelial tumors arising in mice fed LFD-HFD and HFD-LFD showed greater proliferation with BP-3 treatment (Figure 5A) and spindle cell tumors arising in mice fed LFD and LFD-HFD showed greater proliferation with BP-3 treatment (Figure 5B). Both epithelial and spindle cell tumors arising in mice fed HFD-LFD showed reduced proliferation compared to LFD-fed mice. ANOVA (Supplementary Table 1) found that overall dietary effects on proliferation of epithelial tumors were not significant, while BP-3 effects were significant and showed a significant interaction with diet. ANOVA found both significant dietary and BP-3 effects on proliferation of spindle cell tumors, but no interaction between these treatments.

**Figure 6:**
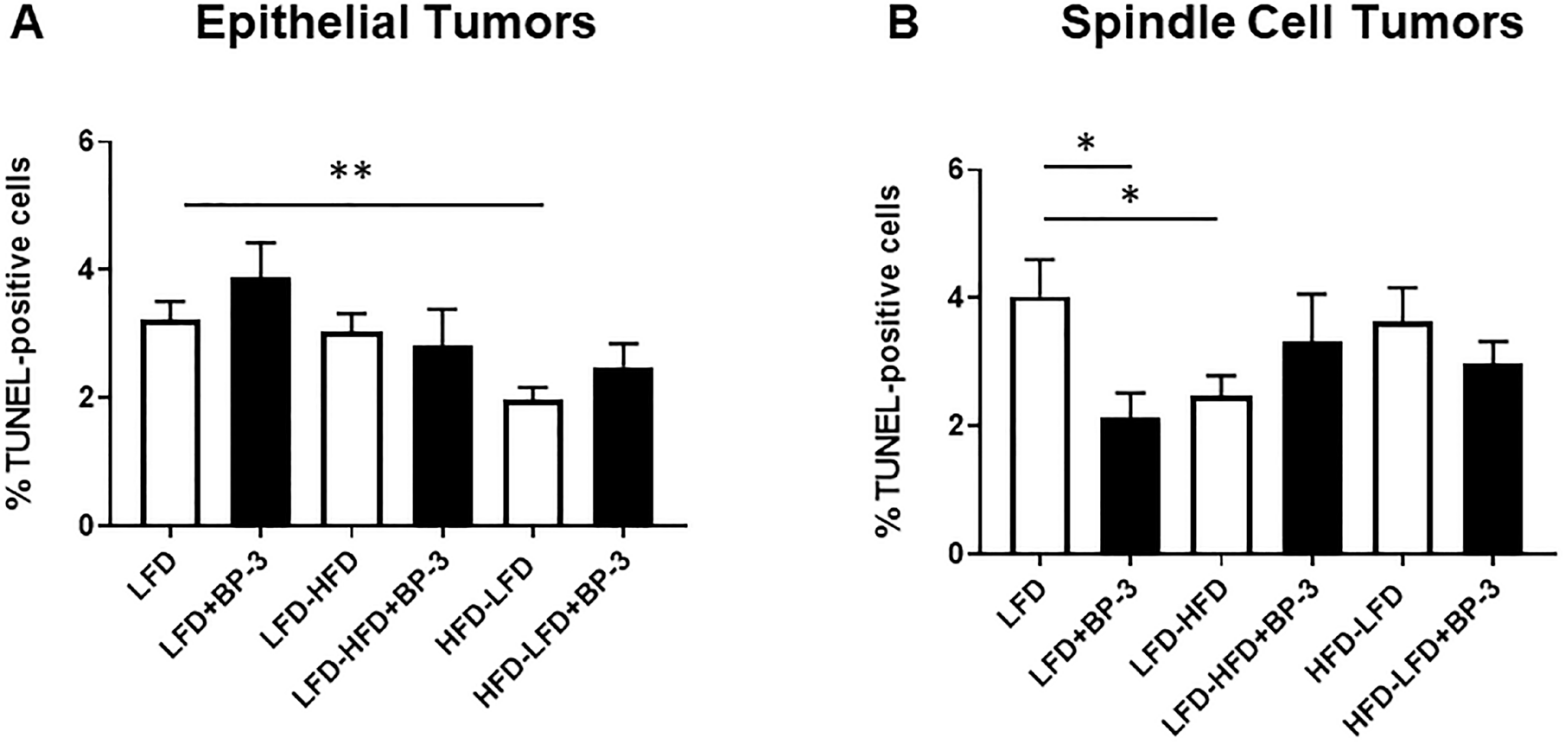
BP-3 treatment decreased apoptosis in spindle cell tumors arising in mice fed LFD. Tumors are grouped by epithelial (**A**) and spindle cell (**B**) histology. Epithelial tumors: LFD (*n* = 8); LFD+BP-3 (*n* = 9); LFD-HFD (*n* = 9); LHF-HFD+BP-3 (*n* = 4); HFD-LFD (*n* = 10); HFD-LFD+BP-3 (*n* = 7). Spindle cell tumors: LFD (*n* = 10); LFD+BP-3 (*n* = 5); LFD-HFD (*n* = 8); LFD-HFD+BP-3 (*n* = 6); HFD-LFD (*n* = 6); HFD-LFD+BP-3 (*n* = 6). The values presented are means +/– SEM. Significance of differences between samples was assessed using an unpaired two-tailed Student’s *t*-test. ^*^ *p* < 0.05; ^**^ *p* < 0.01.

BP-3 did not significantly alter apoptosis, except for spindle cell tumors arising in mice fed LFD (Figure 6). Apoptosis in spindle cell tumors from LFD + BP-3 mice was reduced by half compared to those from LFD mice (Figure 6B). We also observed a reduction of apoptosis in epithelial tumors arising in mice fed HFD-LFD (Figure 6A) and in spindle cell tumors arising in mice fed LFD-HFD compared to LFD (Figure 6B). It is noteworthy that the spindle cell tumors arising in mice fed LFD + BP-3 showed both higher proliferation and lower apoptosis, but longer latency. ANOVA (Supplementary Table 1) found a significant dietary effect on apoptosis in epithelial tumors, but no significance to the effects of diet and BP-3 in spindle cell tumors. However, the interaction between diet and BP-3 was significant for spindle cell tumors, consistent with BP-3 having a significant effect only on tumors arising in mice fed LFD.

**Figure 7:**
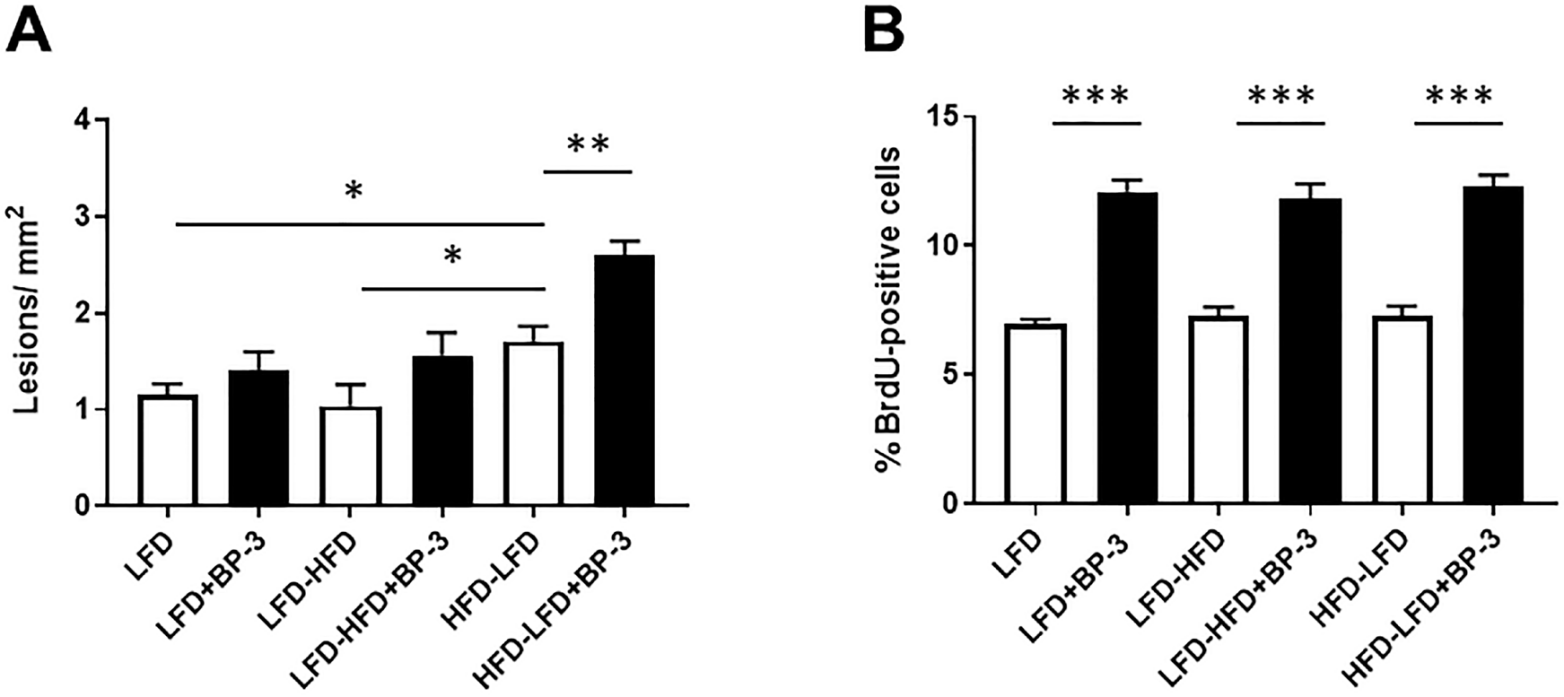
No association between effects of BP-3 on epithelial proliferative lesions, mammary epithelial proliferation, and tumorigenesis. (**A**). Epithelial proliferative lesions are increased by BP-3 treatment in mice fed HFD-LFD. Epithelial proliferative lesions were quantitated in whole mount preparations of the mammary glands of mice at 26 weeks of age across treatment groups. LFD (*n* = 7); LFD+BP-3 (*n* = 8); LFD-HFD (*n* = 7); LFD-HFD+BP-3 (*n* = 7); HFD-LFD (*n* = 7); HFD-LFD+BP-3 (*n* = 7). (**B**) BP-3 treatment increased mammary epithelial proliferation across all dietary regimens. LFD (*n* = 5); LFD+BP-3 (*n* = 5); LFD-HFD (*n* = 7); LFD-HFD+BP-3 (*n* = 5); HFD-LFD (*n* = 5); HFD-LFD+BP-3 (*n* = 5). The values presented are means +/– SEM. Significance of differences between samples was assessed using an unpaired two-tailed Student’s *t*-test. ^*^ *p* < 0.05; ^**^ *p* < 0.01; ^***^ *P* < 0.001.

Having observed increased proliferation with BP-3 treatment in epithelial tumors arising in mice fed LFD-HFD and HFD-LFD and in spindle cell tumors arising in mice fed LFD and LFD-HFD, we examined whether this would be reflected in the number of epithelial proliferative lesions observed in the mammary glands of 26-week old mice, prior to the appearance of palpable tumors, as well as in the proliferation of normal mammary tissue. BP-3 treatment only increased the number of lesions in mice fed HFD-LFD (Figure 7A), while proliferation was increased by BP-3 treatment in all dietary groups (Figure 7B). No association was observed between BP-3 treatment effects on normal cellular proliferation and tumorigenesis, or between BP-3 treatment effects on epithelial proliferative lesions and tumorigenesis. Mice fed HFD-LFD also showed increased lesions compared to mice fed LFD (Figure 7A). ANOVA (Supplementary Table 1) found both significant dietary and BP-3 effects on the number of lesions, although these effects show no significant interaction. This is consistent with BP-3 showing a trend toward increased lesions in all dietary groups, and the HFD-LFD group showing a significantly higher number of lesions compared to the LFD and LFD-HFD groups. ANOVA only found a significant effect for BP-3 treatment on proliferation in mammary glands of 26-week old mice.

**Figure 8:**
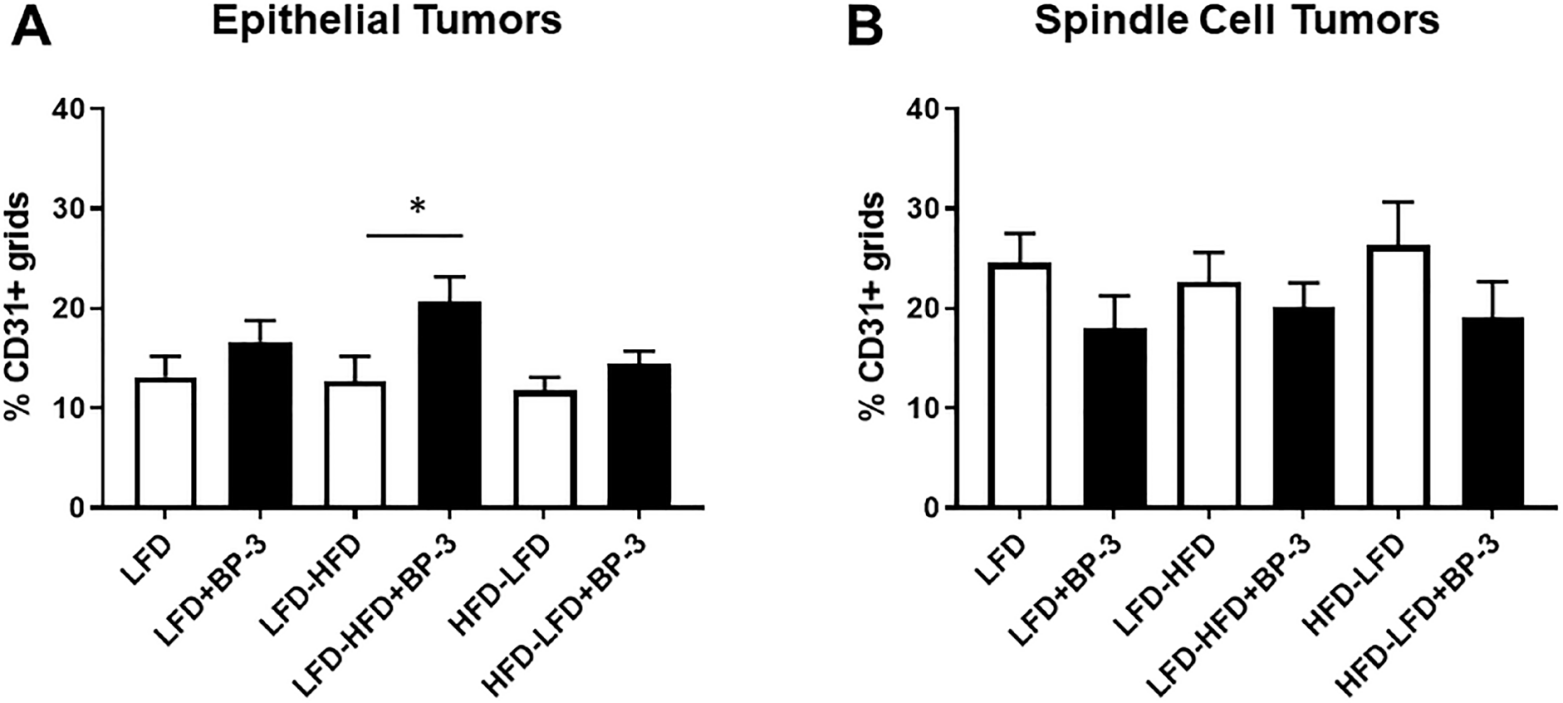
BP-3 treatment increased the vascularization of epithelial tumors in mice fed LFD-HFD. (**A**) Vascularization was assessed in epithelial tumors across the treatment groups by CD31 staining of blood vessels. LFD (*n* = 10); LFD+BP-3 (*n* = 10); LFD-HFD (*n* = 8); LFD-HFD+BP-3 (*n* = 9); HFD-LFD (*n* = 9); HFD-LFD+BP-3 (*n* = 9). (**B**) Vascularization was assessed in spindle cell tumors. LFD (*n* = 10); LFD+BP-3 (*n* = 9); LFD-HFD (*n* = 10); LFD-HFD+BP-3 (*n* = 9); HFD-LFD (*n* = 8); HFD-LFD+BP-3 (*n* = 10). The values presented are means +/– SEM. Significance of differences between samples was assessed using an unpaired two-tailed Student’s *t*-test. ^*^ *p* < 0.05.

Because we found that HFD promoted angiogenesis among epithelial tumors arising in both the *Trp53-null* transplantation model [7] and in the 7,12-dimethylbenz[a]anthracene (DMBA) model [5], we examined the intra-tumoral vascularization of tumors with and without BP-3 treatment using the endothelial cell marker, CD31. Epithelial tumors arising in mice fed LFD-HFD showed increased vascularization in response to BP-3 treatment (Figure 8A). Spindle cell tumors as a group showed increased vascularization compared to epithelial tumors (Figure 8A and 8B; Supplementary Figure 2), as previously reported [7]. ANOVA (Supplementary Table 1) found significant BP-3 effects for both epithelial and spindle cell tumors, consistent with small but statistically insignificant increases in vascularization of LFD and HFD-LFD epithelial tumors, as well as small but statistically insignificant decreases in vascularization of spindle cell tumors with BP-3 treatment.

### Effects on metabolic parameters

Animal weight was followed over the time course of tumorigenesis and BP-3 exposure had no significant impact on body weight (Supplementary Figure 3). BALB/c mice were previously reported to be obesity-resistant when fed HFD [5–8]. In the present study, we observed considerable weight gain among mice fed LFD-HFD. The mice fed LFD-HFD and LFD-HFD + BP-3 were significantly heavier than those fed LFD and LFD + BP-3 by 11 weeks of age, with at least 20% weight gain by 16 weeks of age in (Supplementary Figure 3). While certainly not obesity-resistant in these experiments, the weight gain of mice fed LFD-HFD is not correlated with elevated non-fasting plasma glucose levels (Supplementary Figure 4A), although non-fasting plasma insulin levels were elevated in mice fed LFD-HFD (Supplementary Figure 4B). BP-3 modestly reduced glucose levels in mice fed LFD-HFD and HFD-LFD (Supplementary Figure 4A), and modestly increased insulin levels in mice fed HFD-LFD (Supplementary Figure 4B). ANOVA (Supplementary Table 1) found significant BP-3 effects on glucose levels, consistent with the modest reductions found across dietary groups. ANOVA (Supplementary Table 1) also found significant dietary effects on insulin levels, consistent with modest elevation of insulin across dietary groups. Kaplan-Meier analysis revealed that the weight gain in mice fed LFD-HFD did not significantly alter epithelial tumorigenicity, whether with or without BP-3 treatment (Supplementary Figure 5A and 5B). Segregation of spindle cell tumors between weight groups do not provide an adequate sample size for meaningful analysis. We speculate that this change in weight gain from prior experiments may be the result of housing changes in our mouse facility. Weight gain in mice has been attributed to a shift from sub-thermoneutral to thermoneutral conditions [22].

### Hormonal effects

As BP-3 has known estrogenic and anti-estrogenic properties [9], and our studies herein show BP-3 enhanced estrogen-stimulated mammary proliferation, we examined estrogen levels in plasma from sacrificed tumor-bearing mice (Supplementary Figure 6). We observed a modest, but statistically significant, reduction in estrogen levels by BP-3 treatment of mice fed HFD-LFD. Consistent with slight reductions in estrogen with BP-3 for the other dietary regimes, ANOVA (Supplementary Table 1) found a significant BP-3 effect across all diets.

We also examined the estrus cycle in tumor-bearing mice at the time of tumor collection (Supplementary Figure 7). In the context of the mammary gland, we grouped proestrus together with estrus as periods of growth, and diestrus together with metestrus as periods of regression [23]. In mice fed LFD, there was a statistically significant increase in the proportion of mice in diestrus/metestrus with a complementary decrease in the proportion of mice in proestrus/estrus.

## DISCUSSION

In this study, we showed that long term BP-3 exposure affects the course of *Trp53-null* initiated tumorigenesis in a manner dependent upon both the temporal inclusion of HFD in the feeding regimen of mice and the histopathology of the tumor. BP-3 exposure to mice fed an adult-restricted HFD (LFD-HFD) promoted the incidence of epithelial tumors with increased proliferation and increased vascularization, while decreasing the incidence of spindle cell tumors. Spindle cell tumors occurring in mice fed LFD-HFD with BP-3 exposure also had increased proliferation. In contrast, BP-3 exposure to mice fed a lifelong LFD decreased the incidence of epithelial tumors, and increased the latency of both epithelial and spindle cell tumors. Interestingly, while BP-3 increased the latency of spindle cell tumors in mice fed LFD, those tumors that did occur showed both increased proliferation and a two-fold decrease in apoptosis. Thus, although the latency of spindle cell tumors increased, these tumors displayed two characteristics of higher-grade tumors.

BP-3 exposure to mice fed a pubertally restricted HFD (HFD-LFD) produced no significant change in the incidence of either epithelial or spindle cell tumors compared to mice not treated with BP-3. It should be noted, however, that the brief exposure to HFD between three and ten weeks of age was sufficient to eliminate all protective effects of BP-3 found in mice fed LFD, suggesting puberty as a sensitive window of susceptibility to the effects of BP-3 with HFD. In these studies, our initial examination of BP-3 effects on estrogen-stimulated proliferation in wild type mammary glands only found enhanced proliferation in pubertal mice. This is consistent with the notion of puberty as a particularly sensitive window to environmental toxicants.

In our previous studies, we found that HFD promoted mammary tumorigenesis in both the DMBA-induced tumorigenesis model [5, 6, 8] and in the current *Trp53-null* transplantation model [7]. A pubertal window of susceptibility was observed in these studies and the current study of BP-3 exposure reinforces the finding that puberty is a very sensitive time window for adverse exposures. Regarding BP-3, it will be valuable to eventually explore pubertal versus adult exposure to BP-3 on a constant diet regimen.

The observation of BP-3 promotion of epithelial tumors in mice fed LFD-HFD, while seemingly protecting against spindle cell tumors, raises the question of whether this is an issue of differential response of tumors with differing histopathologies or BP-3 blocking the progression of epithelial tumors to a spindle cell histopathology under conditions of adult HFD. If the latter scenario were the case, one would expect increased latency of spindle cell tumors with BP-3 treatment. This is not the case. How might an LFD-HFD diet regimen favor epithelial tumors over spindle cell tumors? Possible mechanisms might include alteration of the target cell population, alteration of the immune milieu, and alteration of the spectrum of available growth factors. None of these are mutually exclusive. We note that with bilateral transplantation of *Trp53-null* tissue, we cannot exclude the possibility that tumorigenesis in the first gland affects tumorigenesis in the second gland through systemic changes in immune function or secretion of growth factors. While we found that excretion of BP-3 in urine did not differ between diets, suggesting similar dosage, we also cannot exclude the possibility some effects are due to differential retention of BP-3 in mammary fat across the dietary regimens. Nonetheless, BP-3 treatment clearly shows differential effects between the three dietary regimens.

Given the existing literature on BP-3 as an endocrine disruptor, we examined estrogen levels in tumor-bearing mice at their time of sacrifice and found very modest decreases in estrogen levels with BP-3 treatment on all diets (only statistically significant for the HFD-LFD group). Looking further for potential systemic endocrine disruption, we examined the stage of estrus among mice at the time of tumor collection. Only the mice fed LFD showed a significant change in response to BP-3 treatment. They showed an increase in the proportion of mice in the diestrus and metestrus stages of the estrus cycle. These are periods of ductal regression in the mammary glands. It is tempting to speculate that this may, in part, be a factor in the seeming protective effect of BP-3 treatment in mice fed LFD that was observed with Kaplan-Meier analysis. As progesterone induces progression to diestrus, this raises the possibility the BP-3 might exert progestogenic effects in this context. We are aware of some reports of anti- progestogenic effects in the literature [9].

Examination of the mammary glands of 26-week old mice for BP-3 effects on the occurrence of lesions and proliferation were discordant with results from tumors. While BP-3 effects on tumor promotion were most dramatic for epithelial tumors occurring in mice fed a HFD in adulthood (i.e., LFD-HFD), BP-3 was only seen to enhance the number of pre-neoplastic lesions in animals that received a pubertal HFD (i.e., HFD-LFD). This is consistent with our observation that BP-3 only contributed to proliferation in intact mammary glands of mice fed a pubertal HFD. Perhaps, the increase in lesions in HFD-LFD mice reflects this enhanced proliferation during puberty. On the other hand, all mammary glands of 26-week old mice, irrespective of diet, showed enhanced proliferation. This is superficially at odds with our experiments in intact animals that found no proliferative effects in adult mammary glands, irrespective of diet. Perhaps, this discordance reflects the difference between the relatively brief 5-d exposure in our preliminary experiments with intact wild-type mammary glands compared to the lengthy exposure in the mice that comprised the tumorigenesis experiments. Our metabolic studies in BP-3 treated tumor-bearing mice, where BP-3 exposure is long term, suggest modestly increased insulin levels that could plausibly enhance proliferation [24–27]. Another possibility is that differences from wild type tissue reflect altered growth regulation in *Trp53-null* epithelium. Neither rationale is sufficient to explain epithelial proliferation being increased by BP-3 under all diets at 26 weeks of age, while epithelial tumorigenesis as assessed by Kaplan-Meier analysis being promoted by BP-3 only in mice fed an adult HFD. Clearly, tumorigenesis outcomes integrate not only effects intrinsic to the epithelium, but also effects impacting immune status and extrinsic growth factors, to name just two examples. Both are certainly ripe areas for further study.

Unlike several earlier studies that found rather minimal BP-3 activity *in vivo* in rodents with doses higher than those in the current study: 1500 mg/kg BW/d [20]; 1000 mg/kg BW/d [28]; 150 mg/kg BW/d [29], our tumorigenesis experiments showed significant effects at 70 mg/kg BW/d. This dosage elicits levels of BP-3 excretion in urine similar to that observed in humans with heavy topical application of BP-3-containing sunscreen [19]. Furthermore, our initial experiments in wild type BALB/c mice found significant effects on mammary epithelial proliferation at only 7 mg/kg BW/d. Our observations suggest caution in the use of BP-3-containing sunscreens.

## MATERIALS AND METHODS

### Mice

BALB/c *Trp53+/–* breeding mice were obtained from Dr. D. Joseph Jerry (University of Massachusetts, Amherst MA), and *Trp53-null* mice were generated as described [18]. The female *Trp53-null* tissue donor mice were maintained on chow diet before mammary gland collection at eight weeks of age. Wild-type recipient female BALB/c mice were purchased from Charles River (Portage, MI) at 3 weeks of age.

To assess BP-3 stimulation of mammary epithelial proliferation in pubertal wild-type mammary glands, 3-week-old female BALB/c mice were placed on LFD and HFD and, after one week on diet, those that had initiated estrous cycling were ovariectomized (OVX). Recovery was allowed for 3 weeks after OVX to permit complete terminal end bud regression before treatments [30]. To assess BP-3 stimulation of mammary epithelial proliferation in adult wild-type mammary glands, 10-week-old female BALB/c mice were placed on LFD and HFD and, after 3 weeks on diet, were OVX. Recovery was allowed for one week after OVX before treatments. For the long term uniform dosage experiment presented in Figure 1A, mice were fed diets with and without BP-3 (70 mg/kg body weight (BW)) (Spectrum Chemical, New Brunswick, NJ), and then were injected daily for 5 d with saline control or 17-β-estradiol (E2) (1 μg/injection) (Sigma, St. Louis, MO, USA). For the acute dose-response experiment presented in Figure 1B, 3-week-old female BALB/c mice were placed on HFD, and after one week were OVX. Recovery was allowed for 3 weeks after OVX before BP-3 and E2 treatments. Mice were injected daily for 5 d with saline control or E2 (1 μg/injection) and/or BP-3 by oral gavage in vegetable oil (70 mg/kg BW, 7 mg/kg BW, or 0.7 mg/kg BW).

For tumorigenesis experiments, female *Trp53-null* transplanted mice were randomly assigned into six diet groups (see Diets). Food and water were provided *ad libitum*. Mice were housed in a standard laboratory housing environment with a 12:12 h light–dark cycle, at 20 to 24°C with 40 to 50% relative humidity. All mice were sacrificed at estrus. 5-bromo-2′-deoxyuridine (BrdU) (70 μg/g body weight; Sigma-Aldrich, St. Louis, MO, USA) was administered via intraperitoneal injection 2 h prior to sacrifice for analysis of cellular proliferation. All animal experimentation was conducted in accord with accepted standards of humane animal care under guidelines approved by the All University Committee on Animal Use and Care at Michigan State University (AUF #07/17-128-00).

### *Trp53-null* mouse model

Fragments of donor mammary epithelium were collected from female BALB/c *Trp53-null* mice at 8 weeks of age, and transplanted into the cleared inguinal mammary fat pads of 3-week-old female wild type BALB/c mice as previously described [31, 32]. Transplant were performed bilaterally into both inguinal glands. To minimize donor bias from secondary genetic alterations, mammary duct fragments from 4 donor mice were transplanted to recipient mice in each diet group in equal distribution. Body weights and food consumption were monitored weekly. Animals were palpated for tumor development twice a week starting at 13 weeks of age. Tumors were harvested at 1 cm in diameter. At this time, most mice were visually evaluated for stage of estrus. The first tumor occurring in each mouse was removed by survival surgery for analysis and the tumorigenesis time course allowed to continue for the non-tumorous gland. Infrequent tumor recurrence in the first gland was not scored. Portions of tumors and mammary glands were formalin-fixed, paraffin embedded for H&E and immunohistochemistry. Mice were monitored for 500 d, and at termination of the studies, mammary glands were formalin-fixed and processed as whole mounts to evaluate transplantation success rate. Transplantation success rates were 95%, 90%, 92%, 97%, 93%, and 95% for the LFD, LFD+BP-3, LFD-HFD, LFD-HFD+BP-3, HFD-LFD, and HFD-LFD+BP-3 groups, respectively. Wholemount preparations of glands from 26-week-old transplanted mice were scored for the presence of hyperplastic lesions and neoplasia [33]. All lesions and tumors were reviewed and classified, as previously described [34]. Mammary glands that had no epithelium present were excluded from the analysis of tumor incidence.

### Diets

Low fat diet (D11012202; 10% kcal fat) and high fat diet (D11012204; 60% kcal fat) were purchased from Research Diets (New Brunswick, NJ, USA). See Supplementary Table 3 for detailed composition of the diets. For the continuous LFD group, the diet was initiated after transplantation at 3 weeks of age and maintained throughout the studies. For the HFD-LFD and LFD-HFD groups, mice were initially fed one diet from 3 weeks until 10 weeks of age, and then switched to the other diet thereafter. For diets containing BP-3, BP-3 (Spectrum Chemical, New Brunswick, NJ, USA) was compounded into the diets at 0.75 g/kg chow for pubertal animals (3 to 10 weeks of age) and 1.5 g/kg chow for adult animals; the difference in BP-3 between the diets for pubertal and adult animals was intended to compensate for the differences in food consumption and body weight with age.

### BP-3 dosage

We sought a BP-3 dosage below a level found to have carcinogenic effects in mice. A prior study subjecting female B6C3F1 mice to 2 years of BP-3 ingestion at 150 mg/kg BW/d found weakly carcinogenic effects [29]. Our dosage of 0.75 g/kg chow for pubertal animals and 1.5 g/kg chow for adult animals yielded BP-3 consumption of approximately 70 mg/kg BW/d; BP-3 consumption was similar between LFD and HFD, and between pubertal and adult animals. Excretion in mice is divided between urine and feces [35]. To examine BP-3 urine excretion, BALB/c mice were fed LFD or HFD from 3 to 7 weeks of age, and then subjected to a single dose of BP-3 by oral gavage (1.3 mg/100 μL vegetable oil; equivalent to 70 mg/kg BW for an average body weight of 18.2 g). Determination of BP-3 levels in urine was performed by the Division of Laboratory Sciences of the National Center for Environmental Health, Center for Disease Control and Prevention (Atlanta, GA). Greater than 90% of BP-3 excretion occurred by 8 h post BP-3 treatment (Supplementary Figure 1) with no significant difference between diets. In longer-term experiments with an extended period of BP-3 ingestion (Figure 1A), excretion in the urine voided at sacrifice for mice treated with BP-3 plus E2 ranged between 1.0 and 6.1 mg/kg BW with no significant differences between diet or life stage. We compared these values to observations in a small human cohort that was exposed to heavy topical application of sunscreen for 5 d in succession followed by 5 d of urine collection without BP-3 exposure [19]. The total urine excretion in that population averaged approximately 2.3 mg/kg BW, assuming an average European adult body weight of 70.8 kg [36]. Considering the rapid excretion in mice, it is likely that our dosage generates urine excretion on the same order as that observed in heavily exposed humans.

### Immunofluorescence and immunohistochemistry

5 μm tumor sections were deparaffinized and rehydrated, as previously described [5]. For immunofluorescent detection, antigen retrieval was accomplished by autoclaving at 121°C and 15 psi for 20 min in citrate buffer (pH 6.0). For proliferation, BrdU was detected using a mouse monoclonal antibody (1:100; Cat #: RPN202; GE Healthcare, Little Chalfont, Buckinghamshire, UK) with incubation at room temperature for 2 h followed by Alexa 546–labeled goat anti-mouse secondary antibody (1:200; Invitrogen Molecular Probes, Grand Island, NY). Detection of ERα and PR was performed as previously described [5]. ERα was detected with mouse anti-ERα (1:25 in phosphate-buffered saline (PBS)–0.5% Triton X-100; Cat #: NCL-ER-6F11; Novocastra Laboratories, Ltd, Newcastle upon Tyne, UK) and PR was detected with rabbit anti-PR (1:200 in 2% bovine serum albumin in PBS; Cat #: A0092; DAKO, Carpinteria, CA, USA). Immunofluorescent staining was completed with appropriate secondary antibodies. All immunofluorescence sections were counterstained with 4′,6-diamidino-2-phenylindole to visualize nuclei. Images were captured with a Nikon Eclipse TE2000-U fluorescence microscope (Nikon, Inc.) using a 40× objective. At least 1000 cells per tumor or gland were analyzed. Tumors were considered to be PR+ or ERα+ if 10% or more of the total cells counted were PR+ or ERα+, respectively [37].

For immunohistochemical determination of blood vessel density, antigen retrieval was accomplished by boiling at 95–100°C for 20 min in Tris/EDTA buffer (pH 9.0). Endogenous peroxidase activity was blocked by 0.3% H_2_O_2_ in methanol for 30 min. Cluster of differentiation 31 (CD31) was detected with rabbit polyclonal anti-CD31 (1:50 in PBS; Cat #: ab28364; Abcam, Cambridge, MA) at 4°C overnight. This was followed by biotinylated swine anti-rabbit secondary antibody (1:400 in PBS; Cat #: E0353; Agilent Technologies, Santa Clara, CA, USA) for 60 min at room temperature. Biotinylation was detected with ABC reagent (Cat #: PK-7100; Vector Laboratories Inc., Burlingame, CA, USA) and the DAB kit (Cat #: SK-4105; Vector Laboratories Inc., Burlingame, CA, USA). Images were captured using a Nikon Eclipse E400 light microscope (Nikon, Inc., Melville, NY, USA) with a 40× objective lens. The images were overlaid with grids containing 240 squares (324 μm^2^/square). Blood vessel density is expressed as the percentage of CD31-positive squares.

### Terminal deoxynucleotidyl transferase dUTP nick end labeling (TUNEL)

5 μm tumor sections were deparaffinized and rehydrated. TUNEL analysis was performed using the TdT-FragEL DNA Fragmentation Detection Kit (EMD Millipore, Billerica, MA, USA) following the manufacturer’s directions. At least 1000 cells per tumor were analyzed.

### Metabolic parameters and estrogen

Plasma glucose and insulin levels were measured from samples collected at sacrifice from non-fasting tumor-bearing animals, as previously described [5]. Plasma glucose levels were determined by OneTouch UltraMini (LifeScan, Inc., Milpitas, CA, USA) and the insulin levels were determined with the rat/mouse insulin ELISA kit (EMD Millipore), according to the manufacturer’s instructions. Plasma estrogen was measured with the 17-beta-estradiol ELISA Kit (Abcam).

### Statistical analysis

Data were analyzed by two-way analysis of variance (ANOVA) and post-hoc *t*-test, when applicable. The Mantel-Cox test to analyze significance in Kaplan-Meier temporal tumor development, Fisher’s exact test to analyze proportions of spindle versus epithelial tumors, total tumor incidence, and the estrus cycle, non-parametric Mann-Whitney test to analyze tumor latency, 2-way ANOVA, and two-tailed *t*-test are from the GraphPad PRIZM 7.03 software package (San Diego, CA, USA). Results are shown as means ± standard error of the mean (SEM) or ± standard deviation, as noted in figure legends. Differences were considered significant at *p* < 0.05 using statistical approaches as noted in figure legends.

## CONCLUSIONS

These studies reveal significant effects on the course of *Trp53-null* mammary tumorigenesis induced by exposure to benzophenone-3 (BP-3; oxybenzone), a common active ingredient in sunscreens and other personal care products. Utilizing Kaplan- Meier analysis to measure lifetime “survival” of tumor-free mammary glands, we found that BP-3 elicits both promotional and protective effects on mammary tumorigenesis dependent upon dietary regimen and tumor histopathology. However, even in instances where this analysis shows an ostensibly protective effect, other parameters suggest the potential for greater risk. For example, while BP-3 treatment enhances tumor-free survival in mice fed LFD, the spindle cell tumors that do occur display a higher level of proliferation and a lower level of apoptosis, properties associated with a poorer prognosis in human cancers. We also found that pubertal exposure to HFD was sufficient to counter the ostensibly protective effect of BP-3 in mice fed LFD. Thus, our studies contribute to an existing literature suggesting that puberty is a critical window of susceptibility for later mammary tumorigenesis.

Taken together, these findings suggest that BP-3 exposure may have adverse consequences in mammary tumorigenesis. They point to a need for further studies of BP-3 in both animal models and humans as a potential risk factor in breast cancer. They also point to the more general need to evaluate endocrine disrupting chemicals in the context of varying diets. Future studies are needed to identify the mechanistic basis for BP-3 effects on mammary tumorigenesis and how dietary fat interacts with BP-3 to alter outcomes.

## SUPPLEMENTARY MATERIALS



## Abbreviations

ANOVA: Analysis of variance
BP-3: benzophenone-3
BrdU: 5-bromo-2′-deoxyuridine
BW: body weight
CD31: cluster of differentiation 31
DMBA: 7:12-dimethylbenz[a]anthracene
E2: 17-β-estradiol
EDC: endocrine disrupting chemical
ER: estrogen receptor
H&E: hematoxylin and eosin
HFD: high saturated animal fat diet
LFD: low fat diet
OVX: ovariectomized
PBS: phosphate-buffered saline
PR: progesterone receptor
SEM: standard error of the mean
Trp53: transformation-related protein 53
TUNEL: Terminal deoxynucleotidyl transferase dUTP nick end labeling
