# Supplementary material for: Benzophenone-3 promotion of mammary tumorigenesis is diet-dependent
Source: Oncotarget. 2020 Dec 1;11(48):4465–78. doi: 10.18632/oncotarget.27831 (PMC7721615; doi:10.18632/oncotarget.27831)
Supplement: Supplementary file 1 [file oncotarget-11-4465-s001.pdf]

Benzophenone-3 promotion of mammary tumorigenesis is diet-dependent

SUPPLEMENTARY MATERIALS

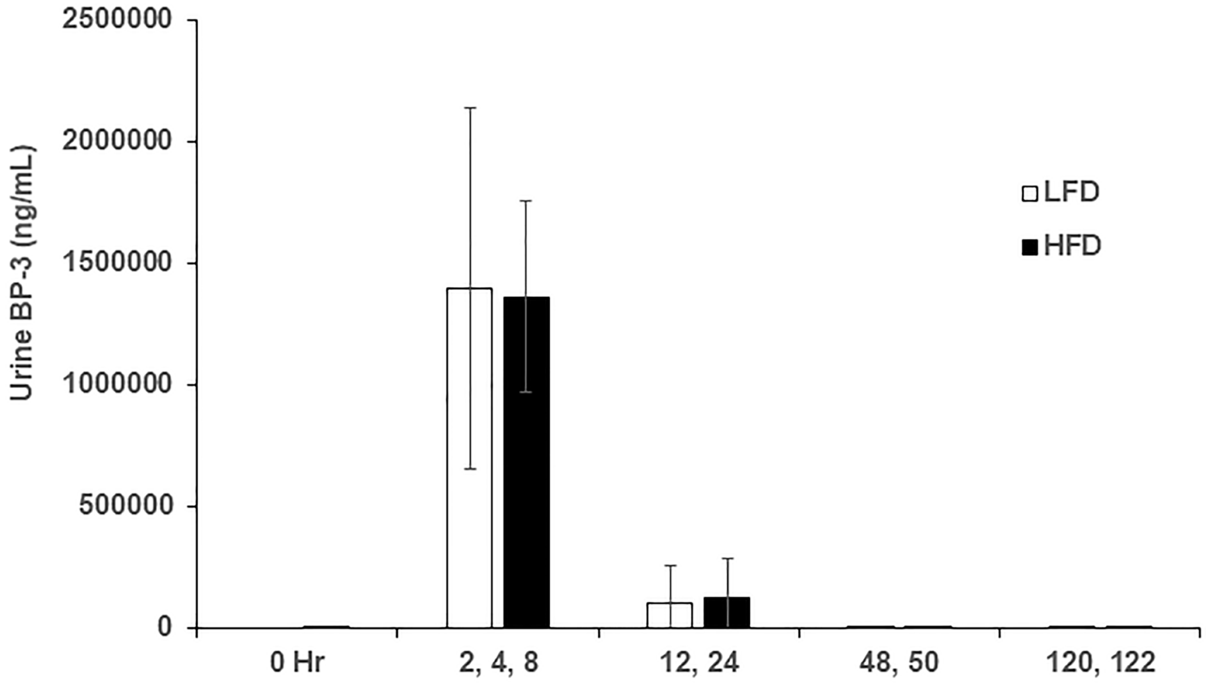

Supplementary Figure 1: BP-3 excretion in urine is rapid in mice. A time course of urine excretion in mice showed that greater than 90% of BP-3 excretion occurred by 8 h post BP-3 treatment. BALB/c mice were fed LFD or HFD from 3 to 7 weeks of age, and then subjected to a single dose of BP-3 by oral gavage. The 2-, 4-, and 8-h, and 12- and 24-h time points were each pooled for analysis because individual mice (n = 4) did not produce urine at some time points. The 48- and 50-h, and 120- and 122-h time points represent samples collected at one or the other time point for each mouse. The values presented are means +/– standard deviation.

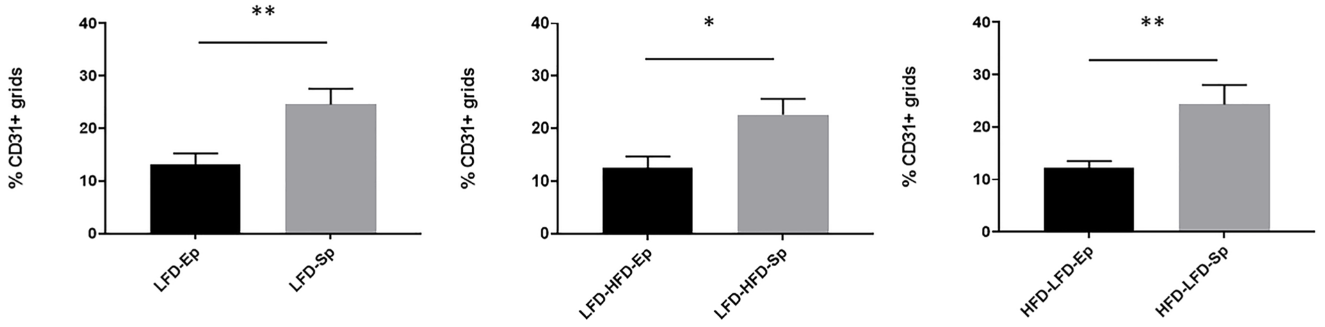

Supplementary Figure 2: Spindle cell tumors are more vascularized than epithelial tumors. Vascularization was compared between epithelial tumors and spindle cell tumors within the same dietary group by CD31 staining of blood vessels. The tumor samples are the same as those analyzed in Figure 8. Epithelial tumors from mice fed LFD (LFD-Ep); spindle cell tumors from mice fed LFD (LFD-Sp); epithelial tumors from mice fed LFD-HFD (LFD-HFD-Ep); spindle cell tumors from mice fed LFD-HFD (LFD-HFD-Sp); epithelial tumors from mice fed HFD-LFD (HFD-LFD-Ep); spindle cell tumors from mice fed HFD-LFD (HFD-LFD-Sp). The values presented are means +/– SEM. Significance of differences between samples was assessed using an unpaired two-tailed Student's t-test. $^{*}p < 0.05$; $^{**}p < 0.01$.

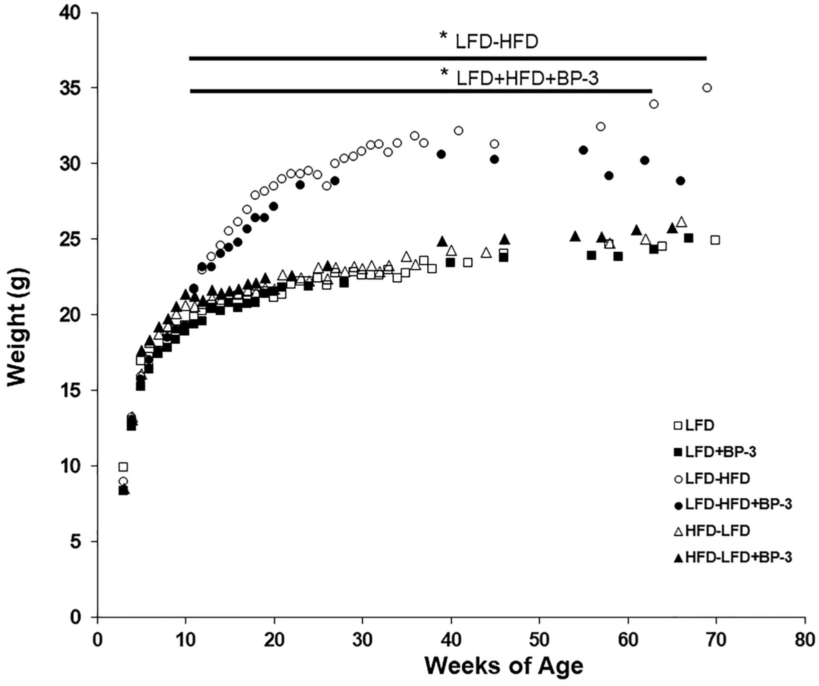

Supplementary Figure 3: BP-3 treatment does not affect body weight. While BP-3 did not affect body weight, mice fed an adult restricted HFD (LFD-HFD) gained weight compared to mice fed lifelong LFD (LFD) and mice fed a pubertally-restricted HFD (HFD-LFD). Significance of differences between mice fed LFD-HFD versus LFD and mice fed LFD-HFD+BP-3 versus LFD+BP-3 was assessed using an unpaired two-tailed Student's t-test. $^{*}p < 0.05$.

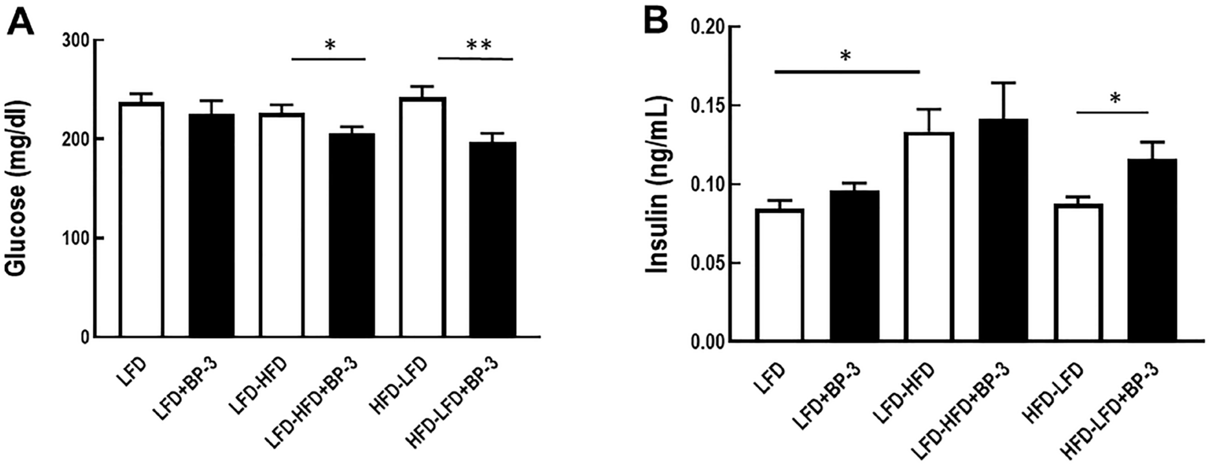

Supplementary Figure 4: BP-3 treatment and weight gain have modest effects on glucose and insulin levels. (**A**) Glucose levels were measured in the plasma of non-fasting mice across treatment groups. (**B**) Insulin levels were measured in the plasma of non-fasting mice across treatment groups. The values presented are means +/– SEM. Significance of differences between samples ($n = 5$ for each treatment group) was assessed using an unpaired two-tailed Student's t-test. $^{*}p < 0.05$; $^{**}p < 0.01$.

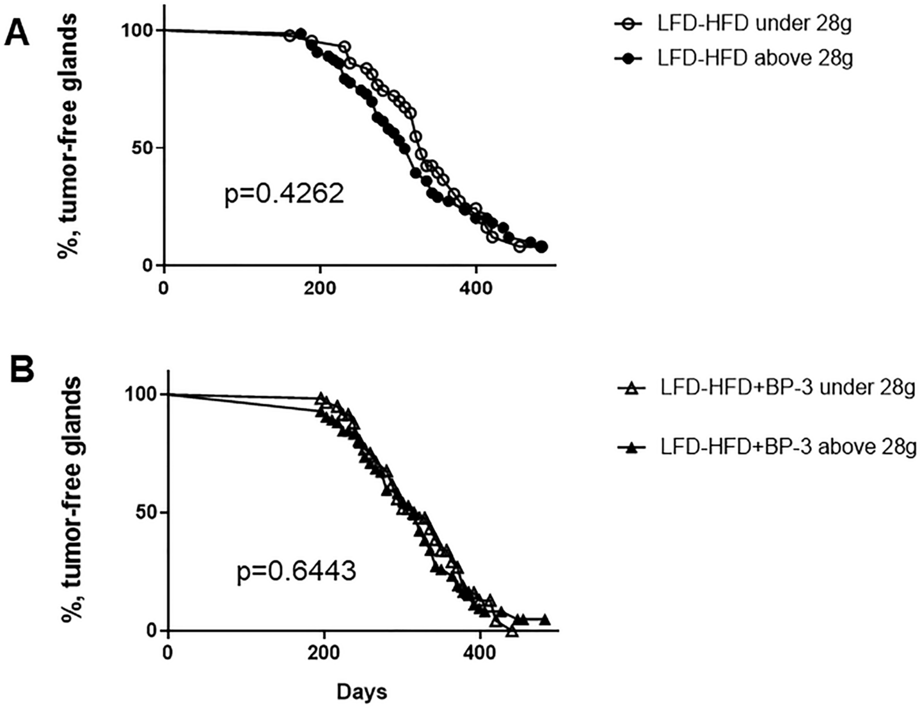

Supplementary Figure 5: Weight gain has no impact on tumorigenesis, as assessed by Kaplan-Meier analysis. (**A**) Mice weighing above and below 28 g were compared among those fed LFD-HFD. (**B**) Mice weighing above and below 28 g were compared among those fed LFD-HFD+BP-3, Significance of differences between plots was assessed by the log-rank Mantel–Cox test. Differences were not significant. p values are displayed on the graphs.

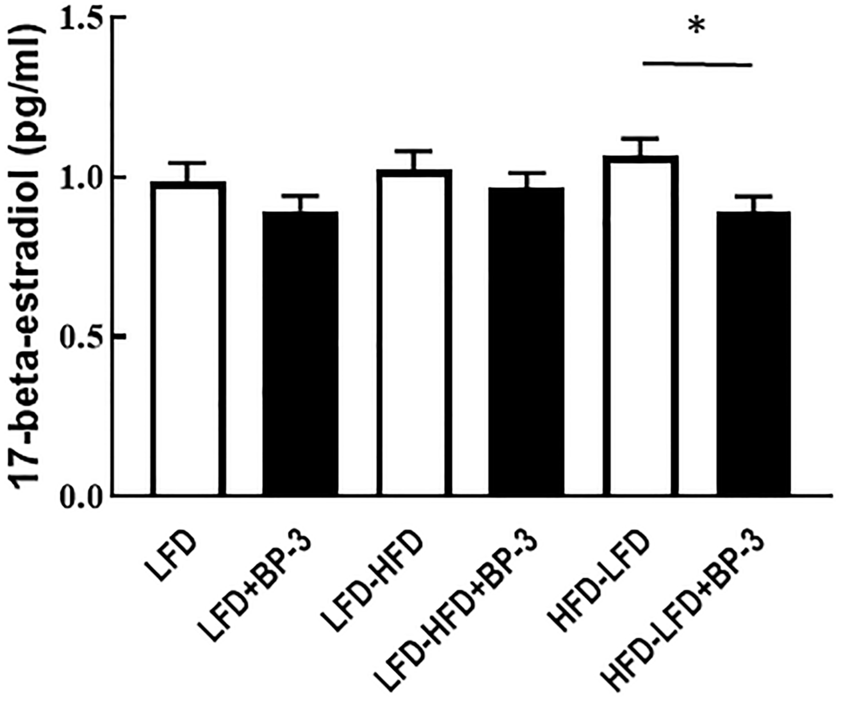

Supplementary Figure 6: BP-3 treatment has modest effects on estrogen levels. 17-β-estradiol was measured in plasma collected at the time of sacrifice from tumor-bearing mice. The values presented are means +/– SEM. Significance of differences between samples (n = 5 for each treatment group) was assessed using an unpaired two-tailed Student's t-test. $^{*}p < 0.05$.

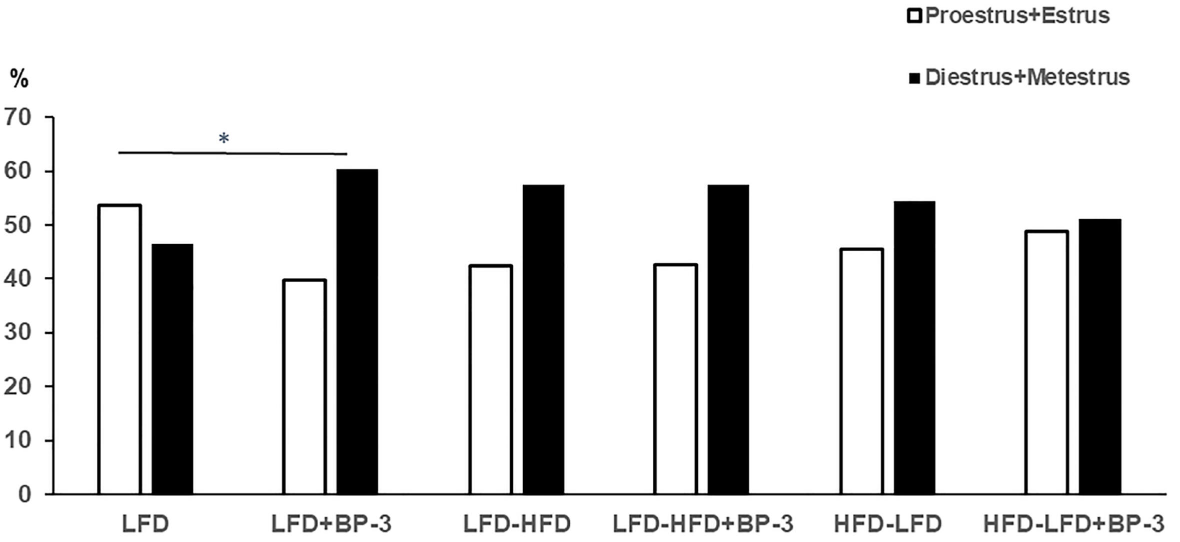

Supplementary Figure 7: BP-3 treatment increased the proportion of mice in diestrus and metestrus compared to those in proestrus and estrus in mice fed LFD. Estrus stage was determined at the time of tumor collection. LFD (n = 112); LFD+BP-3 (n = 126); LFD-HFD (n = 87); LFD-HFD+BP-3 (n = 122); HFD-LFD (n = 99); HFD-LFD+BP-3 (n = 123). Significance of differences between treatment groups was assessed using Fisher's Exact Test. $^{*}p < 0.05$.

Supplementary Table 1: ANOVA of the effects of diet and BP-3 treatment in epithelial tumors, spindle cell tumors, mammary tissue, and plasma, and the interaction of diet with BP-3 treatment

Effect	Tissue Types	Diet	BP-3	Interaction
Latency	Epi	0.0060	NS	NS
	Spindle	0.0126	NS	NS
Proliferation	Epi	NS	< 0.0001	< 0.0001
	Spindle	0.0141	0.0087	NS
TUNEL	Epi	0.0032	NS	NS
	Spindle	NS	NS	0.047
Lesions 26 weeks	Mammary	< 0.0001	0.0002	NS
Proliferation 26 weeks	Mammary	NS	< 0.0001	NS
CD31	Epi	NS	0.0061	NS
	Spindle	NS	0.0490	NS
Glucose	Plasma	NS	0.0035	NS
Insulin	Plasma	0.0009	NS	NS
Estrogen	Plasma	NS	0.0138	NS

Two-way ANOVA was performed on data derived from epithelial (Epi) and spindle cell tumors (Spindle), mammary tissue (Mammary), and plasma. Probability values are entered where significant ($p < 0.05$). For values not reaching significance, NS is entered.

Supplementary Table 2: Proportion of ER+/PR+ tumors by diet treatment and histopathology

Diet	Tumor Type	ER+PR+/Total
LFD	Epithelial	1/10
	Spindle Cell	0/10
LFD+BP-3	Epithelial	1/11
	Spindle Cell	1/10
LFD-HFD	Epithelial	1/10
	Spindle Cell	0/8
LFD-HFD+BP-3	Epithelial	0/10
	Spindle Cell	1/8
HFD-LFD	Epithelial	1/10
	Spindle Cell	0/10
HFD-LFD+BP-3	Epithelial	0/10
	Spindle Cell	0/10

Supplementary Table 3: Diet composition

**Ingredients (g/100 g)**		**Low Fat Diet**	**High Fat Diet**
Fat	Corn Oil	2.369	16.1498
	Lard	1.8957	31.6537
Carbohydrate	Corn Starch	54.407	8.888
	Maltodextrin	11.848	16.1498
Protein	Casein	18.987	25.8397
	L-cystine	0.2843	0.3876
Fiber	Cellulose	4.7393	6.4599
Vitamins	Vitamin Mix V10001	0.9479	1.2919
	Choline Bitartrate	0.1896	0.2584
Minerals	Mineral Mix S10026	0.9479	0.1286
	DiCalcium Phosphate	1.2322	1.6795
	Calcium Carbonate	0.5213	0.7106
	Potassium Citrate, 1 H20	1.5639	2.1318
**Energy**			
kcal density/g		3.8	5.2
% kcal	Fat	10	60
	Carbohydrate	70	20
	Protein	20	20
